# A Century of Shifting Native Species‐Accumulation Curves Reveals Long‐Term Biodiversity Loss

**DOI:** 10.1111/ele.70429

**Published:** 2026-06-15

**Authors:** Kaixuan Pan, Leon Marshall, Jacobus C. Biesmeijer, Geert R. de Snoo

**Affiliations:** ^1^ Naturalis Biodiversity Center Leiden the Netherlands; ^2^ Institute of Environmental Sciences Leiden University Leiden the Netherlands; ^3^ Netherlands Institute of Ecology (NIOO‐KNAW) Wageningen the Netherlands

**Keywords:** biodiversity conservation, biodiversity patterns, biodiversity recovery, ecosystem restoration, forests, GBF targets, grasslands, long‐term biodiversity change, Natura 2000, reverse biodiversity loss

## Abstract

The species‐area relationship is a cornerstone of biodiversity theory and conservation. Yet, its temporal stability remains largely untested. Using nearly a century of disjunct vegetation‐plot data from the Netherlands, we assess changes in the species‐area relationship by constructing species‐accumulation curves (SACs). We show that SACs have flattened significantly over time, indicating widespread biodiversity decline driven by habitat degradation and spatial homogenization, even within protected areas. Although grassland biodiversity has rebounded, forest biodiversity continues to decline, suggesting a greater extinction debt. These findings provide unprecedented evidence that biodiversity patterns captured by SACs shift over time. They also reveal a critical limitation of conservation strategies that rely solely on protected area expansion. As global biodiversity goals aim to halt biodiversity loss by 2030, our results emphasise the need for adaptive conservation strategies that integrate area‐based protection with large‐scale habitat restoration and improved ecosystem management.

## Graphical abstract

Using nearly a century of vegetation‐plot data across the Netherlands, we show that species‐area relationships have significantly flattened over time, meaning that larger areas are now required to support the same level of species richness as the historical baseline. This shift reflects widespread biodiversity decline and biotic homogenization. While grassland biodiversity has partially recovered since the 1990s, forest biodiversity continues to decline. Together, these findings suggest that expanding protected areas alone will be insufficient to halt biodiversity loss without concurrent restoration of habitat.

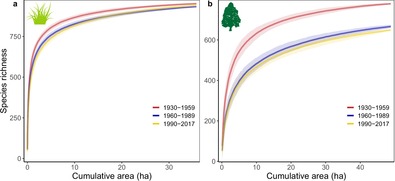

## 1 Introduction

Biodiversity loss is one of the most pressing challenges of the Anthropocene, driven largely by habitat destruction and degradation (Dirzo et al. 2014; Ceballos et al. 2015; Jaureguiberry et al. 2022; Keck et al. 2025). Conservation science must not only document these changes but also test and refine the theories underpinning conservation strategies to ensure their effectiveness (Grant et al. 2019). Although large‐scale biodiversity assessments provide crucial insights into species decline and recovery (Finderup Nielsen et al. 2019; Eichenberg et al. 2020; Jandt et al. 2022), they are often weakly linked to the theoretical foundations and policy frameworks needed to guide effective conservation action. Addressing this gap is critical for developing strategies that are both scientifically sound and practically viable (Laurance et al. 2012; Godet and Devictor 2018).

A cornerstone of macroecology and conservation is the species‐area relationship, which describes how species richness increases with the area sampled (Ugland et al. 2003). This principle, often visualized through species accumulation curves (SACs), has long been used to justify the expansion of protected areas, under the assumption that larger reserves support more biodiversity (Scheiner 2003; Matthews et al. 2016). In response, global conservation frameworks, such as the post‐2020 Global Biodiversity Framework (GBF), have set ambitious targets to increase protected area coverage to 30% by 2030 (CBD 2022). However, the large‐scale temporal dynamics of species‐area relationships and SACs remain poorly understood, despite rapid environmental changes driven by climate shifts, land‐use transformation, pollution and other anthropogenic pressures (Dirzo et al. 2014; Ceballos et al. 2015; Jaureguiberry et al. 2022; Keck et al. 2025). If these relationships shift, the implications for conservation policy are profound, necessitating a revaluation of how protected areas are designed and managed.

Despite widespread efforts to establish protected areas, few studies have rigorously assessed their long‐term effectiveness in preventing biodiversity loss (Soares‐Filho et al. 2010; Nolte et al. 2013; Geldmann et al. 2019). Most existing evaluations rely on indirect indicators such as land cover change, rather than direct biodiversity measures like long‐term species surveys (Santangeli et al. 2023; Li et al. 2024). Additionally, research has disproportionately focused on birds and mammals, leaving plant biodiversity underrepresented in assessments of conservation impact (Santangeli et al. 2023; Williams et al. 2022; Wauchope et al. 2022; Justin Nowakowski et al. 2023). Given the global push to expand protected areas, it is crucial to rigorously quantify their effectiveness across different taxa and ecosystems. Identifying the conservation strategies that have demonstrably improved biodiversity, and understanding why, remains a major scientific and policy priority (Rodrigues and Cazalis 2020).

In this study, we analysed nearly a century's worth of vegetation‐plot records, encompassing over 600,000 plots across multiple time series and diverse regions in the Netherlands, to examine long‐term shifts in species‐area relationships using species‐accumulation curves constructed from spatially disjunct plots, following the conceptual framework described by Smith (2010). To ensure comparability over time, we standardised plot placement and sampling density, controlling for artefacts arising from non‐contiguous plot networks (Smith 2010). By assessing how native species richness accumulates with sampled areas, we evaluated whether the species‐accumulation curves changed over time and whether protected areas have succeeded in maintaining biodiversity.

We tested two key hypotheses: (1) The species‐accumulation curve has shifted over time, reflecting long‐term biodiversity changes and (2) these temporal trajectories of the species‐accumulation curve differ between protected and unprotected areas, with species‐accumulation curves remaining stable inside protected areas, consistent with long‐term protection efforts, whereas shifting outside them. This extensive dataset provides a rare opportunity to evaluate how species‐accumulation relationships evolve over decades and to rigorously assess the long‐term performance of protected areas. Our findings offer new evidence to inform conservation planning and highlight the need for adaptive strategies that integrate area‐based protection with large‐scale habitat restoration and improved ecosystem management.

## 2 Methods

Our analytical workflow is summarized in Figure S1, which outlines the full sequence of data processing, spatial standardization, and statistical analysis used to quantify temporal changes in species‐accumulation patterns.

### 2.1 Plant Plot Data

To analyse species‐accumulation curves, which reflect how species richness increases with the cumulative area of sampled plots, in (semi‐)natural habitats, we utilized data from the Dutch Vegetation Database, which includes over 10 million observations across 675,521 plots (Hennekens 2018). These plots, sampled in various (semi‐)natural habitats throughout the Netherlands since 1930, document complete vascular plant species compositions. We focused on plots sized between 1 and 100 m^2^ and those ranging from 100 to 1000 m^2^, as these sizes are traditionally used in grassland and forest studies by European plant sociologists (Westhoff et al. 1978; Večeřa et al. 2021; Willner and Faber‐Langendoen 2021). Only plots with precise geographic coordinates were included in our analysis. For temporal comparisons, we divided the dataset into three 30‐year periods: 1930–1959, 1960–1989 and 1990–2017. These periods correspond to a historical baseline, a phase of rapid land‐use intensification and habitat loss, and a more recent period characterized by increased conservation efforts and reduced environmental pressures, respectively (van Veen et al. 2008).

### 2.2 Natura 2000 Protected Areas

To assess the effectiveness of protected areas in conserving plant biodiversity over time, we mapped all plot records to Natura 2000 areas in the Netherlands. Natura 2000 is a network of protected areas across Europe aimed at biodiversity conservation. Boundaries for these areas were obtained from the European Commission (https://www.eea.europa.eu/), and we classified plots as ‘protected’ if they were designated as such at any time before, during or after sampling, consistent with the approach of van Klink et al. (2020). This classification assumes that plots later designated as protected were already of high ecological value. Spatial analysis was conducted using the ‘st_intersection’ function from the ‘sf’ package (version 1.0‐19) (Pebesma 2018; Pebesma and Bivand 2023).

### 2.3 Plant Groups

Our analysis focused on species‐level data, excluding any records not identified to the species level to ensure taxonomic accuracy. We analysed plant richness patterns for native species only, with species origin determined using the Red List of Vascular Plants of the Netherlands (Sparrius et al. 2014), ensuring dataset reliability.

### 2.4 Spatial Standardization of Plot Distributions Across Time Periods

Vegetation plots sampled across different time periods varied substantially in spatial distribution, density, size and inter‐plot distances, factors that could confound temporal comparisons of species accumulation curves by conflating area effects with beta‐diversity turnover (Smith 2010). To ensure valid temporal comparisons, we implemented a rigorous spatial standardization procedure controlling for these confounding factors.

*Step 1. Spatial stratification* via *k‐means clustering*: For each habitat type (grasslands: 1–100 m^2^; forests: 100–1000 m^2^) and protection status (Natura 2000 protected and unprotected areas), we delineated spatial regions using k‐means clustering. All plots from the three time periods (1930–1959, 1960–1989 and 1990–2017) were pooled, and their geographic coordinates were subjected to k‐means clustering with a maximum of 100 iterations, partitioning each dataset into 50 spatial clusters (Figure S2: national extent; Figure S5: protected and unprotected subsets). This cluster number was selected to balance spatial resolution with adequate sample size per cluster, ensuring each region contained sufficient plots from multiple periods for robust sampling while representing distinct geographic areas across the Netherlands.

*Step 2. Within‐cluster plot selection with constraint matching*: For each spatial cluster and time period, we calculated three key metrics to characterise the sampling design. First, we counted the number of plots within each cluster‐period combination. Second, we quantified spatial clustering by calculating mean nearest‐neighbour distance: for each plot, we determined the mean Euclidean distance to its 10 nearest neighbours within the same cluster and period, then averaged this value across all plots in the cluster. Third, we calculated the arithmetic mean of plot areas within each cluster‐period combination.

*Step 3. Determination of standardization targets*: To ensure comparability across time periods, we established target values for each cluster based on the most restrictive period. For plot counts, we used the minimum number of plots available across the three periods within each cluster, ensuring we could sample equal numbers from each period. For nearest‐neighbour distance, we selected the maximum mean distance observed across periods, ensuring that standardization did not force any period to be sampled more densely than it was originally. Similarly, for plot size, we used the maximum mean size observed across periods to prevent sampling at finer spatial grain than the original data allowed. This conservative approach, using minimum plot counts but maximum distance and size, standardizes all periods to match the least restrictive sampling configuration (most spread‐out plots, largest plot areas) while constraining sample size to the most restrictive period. We retained only clusters with data in all three time periods, ensuring truly comparable regional sampling and eliminating the potential bias introduced by clusters sampled in only one or two periods.

*Step 4. Constrained random sampling within clusters*: For each cluster and time period, we employed an iterative constrained sampling algorithm to select plots matching the target specifications. Within each cluster‐period combination, we randomly selected the target number of plots without replacement, then calculated the mean nearest‐neighbour distance and mean plot size for this sample. These sampled values were compared to the cluster‐specific targets using relative deviations, defined as the absolute difference between sampled and target values divided by the target value. If both the distance deviation and size deviation were within 10% of their targets, the sample was accepted immediately. Otherwise, we calculated a composite score as the sum of distance and size deviations, tracking the sample with the lowest score across iterations. This process was repeated up to 100 times per cluster‐period combination, with the algorithm accepting the first sample meeting tolerance criteria. The 10% tolerance allowed natural variation in continuous variables while ensuring comparability across periods and preventing situations in which no sample could simultaneously satisfy multiple target constraints.

*Step 5. Ensuring equal regional coverage across periods*: After sampling within each cluster‐period combination, we identified clusters successfully sampled in all three time periods. Clusters missing data from any period were excluded from all periods to ensure balanced comparisons. This common‐regions filtering was critical for eliminating sampling artefacts: if a cluster was sampled only in 1930–1959 and 1960–1989 but not 1990–2017, including it would bias temporal comparisons by conflating genuine biodiversity changes with differences in spatial coverage. The final standardized datasets comprised plots from the same set of clusters across all three periods, with equal plot counts per cluster within each period, though different clusters contributed different numbers of plots based on their respective minimum availability across periods.

*Step 6. Validation of standardization quality*: We quantitatively verified whether standardization successfully achieved comparability across time periods using Kruskal‐Wallis tests. By design, plot counts were equal across periods (Figure S3: national extent; Figure S6: protected and unprotected subsets). The results also show that inter‐plot distances and plot sizes were comparable across periods (Figure S4: national extent; Figure S7: protected and unprotected subsets), with most regions displaying the same or similar values across time. This demonstrates that both inter‐plot spacing and plot size were successfully standardized across periods. We hypothesised that if mean nearest‐neighbour distances and mean plot sizes did not differ significantly among periods or, in cases where significant differences occurred, did not show the same temporal pattern as the species‐accumulation curves, then the observed temporal trends in species richness would reflect real ecological change rather than residual sampling bias. Conversely, if mean nearest‐neighbour distances and plot sizes differed significantly among periods and showed the same temporal trend as the species‐accumulation patterns, the richness trends would likely result from sampling artefacts. Our validation results (Figures S3 and S4: national extent; Figures S6 and S7: protected and unprotected subsets) support our hypothesis and show that temporal shifts in species accumulation curves are not explained by residual sampling artefacts but instead reflect genuine ecological changes over time.

All species accumulation curves (rarefaction analyses) and comparison presented in the main text were conducted exclusively on spatially standardized datasets. This ensures that observed temporal shifts in species‐accumulation patterns across the whole Netherlands, inside and outside protected areas, reflect genuine ecological changes rather than artefacts of sampling design.

### 2.5 Species Accumulation Curves and Rarefaction Analysis

We divided the plant plot data into three time periods: [1930–1959], [1960–1989] and [1990–2017]. For each period, we constructed species‐accumulation curves for native species by randomly resampling plots and cumulatively increasing the area of plots. These curves represent how species richness accumulates as more disjunct (non‐contiguous) plots are added to the sample (Smith 2010). To evaluate changes in species‐accumulation patterns over time, we calculated 95% confidence intervals for each period based on the distribution of resampled data.

We compared species‐accumulation patterns between protected and unprotected areas to understand how protection status influences biodiversity over time. For each period, species‐accumulation curves were constructed by randomly resampling plots within both protected and unprotected areas. Given that protected areas are designed to conserve species diversity, we hypothesised that species‐accumulation curves would remain stable over time in protected areas, while they might decline in unprotected areas.

All statistical analyses were performed in R version 4.3.1 (R Core Team 2023).

## 3 Results

### 3.1 Nationwide Trends in Species‐Accumulation Patterns

**FIGURE 1:**
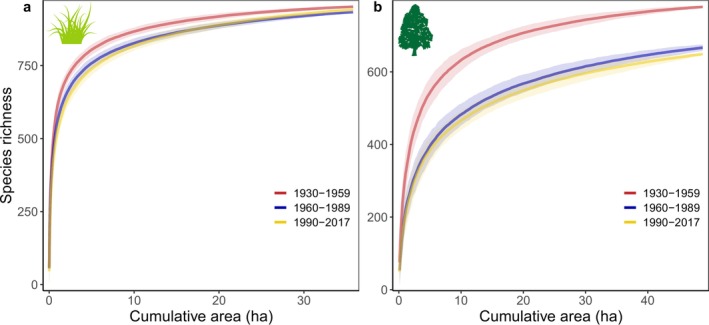
Species‐accumulation curves illustrating native species richness in relation to cumulative sampled area from disjunct plots across grasslands (a) and forests (b). (a, b) Curves show how species richness accumulates as more plots are added to the sample, with total sampled area on the *x*‐axis, for grasslands (1–100 m^2^; a) and forests (100–1000 m^2^; b). Shaded areas indicate 95% confidence intervals.

To assess long‐term changes in biodiversity patterns, we compared SACs for vascular plants across three historical periods. Our analysis reveals that the SAC for 1990–2017 lies consistently below the historical baseline (1930–1959), showing both lower asymptotic richness and shallower slope (Figure 1). Together, these patterns indicate that fewer species have accumulated across comparable sampling extents in recent decades, reflecting fundamental changes in plant species over time.

These shifts in SACs can arise from multiple mechanisms, including reduction in total species richness, altered β diversity, changes in species range sizes or configurations. The lower asymptotes of recent SACs directly reflect a decline in total species richness at the national scale. In addition, the reduced slopes of SACs (Figure 1), together with flatter distance‐decay relationships (Figure S8c–f), indicate a decline in spatial turnover, meaning that communities have become more similar to one another across space. Consequently, detecting the same number of species now requires sampling across more locations.

These patterns are supported by a concurrent decline in mean local (α) diversity across periods (Figure S8a,b), suggesting that biodiversity loss has occurred both locally and regionally. The combined decline in total richness, α diversity at plot scale, and spatial turnover suggests increasing biotic homogenization and decreasing local biodiversity, likely driven by the loss and contraction of some species, particularly habitat specialists, and the spread of generalists. Such trends are consistent with long‐term habitat degradation and environmental change documented across the Netherlands, and are supported by evidence of biodiversity homogenization across Europe (Carvalheiro et al. 2013). However, without explicit information on species' full geographic ranges and their shapes, it is not possible to directly show how species range size and configuration affect the SACs over time, and disentangle changes in range size from changes in range configuration. Overall, the observed decline in alpha diversity and the reduction in beta diversity (plot‐level turnover) together suggest that long‐term biodiversity loss has been driven predominantly by habitat degradation and homogenization of species communities, rather than by habitat loss alone. Species richness peaked during 1930–1959 in both grassland and forest ecosystems (Figure 1), underscoring the importance of habitat quality and heterogeneity in sustaining biodiversity (Chase et al. 2020; Díaz et al. 2019).

Although plant biodiversity declined after the 1930s, a partial recovery of grassland biodiversity was observed by the 1990s, though not to the levels recorded in 1930–1959 (Figure 1a). This suggests that while biodiversity is rebounding, it has yet to return to its historical level (1930–1959). This partial recovery is reflected in increasing total richness and spatial turnover, despite continued declines in local α diversity (Figure S8), suggesting that recovery remains constrained by persistent habitat degradation.

From a conservation perspective, these results suggest that maintaining historical levels of biodiversity may require attention not only to total habitat extent, but also to habitat quality, as well as the spatial configuration and heterogeneity of habitats across landscapes in order to increase both alpha and beta diversity and lead to overall biodiversity conservation.

Notably, forest ecosystems, in particular, experienced a dramatic decline in biodiversity post‐1960s, followed by a slow but continued reduction since the 1990s (Figure 1b). This decline is evident in lower SAC asymptotes and sustained declines in both α and β diversity (Figure S8). These patterns may reflect the limited adaptive capacity of long‐lived forest species to rapid environmental changes, such as climate change and increased nitrogen deposition (Bertrand et al. 2016; Song and Zhu 2024). In contrast, grassland ecosystems, dominated by short‐lived species, may exhibit more rapid adaptation to climate change and other stressors (Zhu et al. 2024), as well as faster recovery dynamics (Figure 1a).

Overall, species‐accumulation patterns have shifted markedly over the past century. Although grassland biodiversity shows signs of recovery, altered SAC shapes indicate that overall biodiversity remains lower. From a conservation perspective, these results emphasise that maintaining or restoring biodiversity requires not only sufficient protected area coverage but also improvements in habitat quality and spatial heterogeneity. This underscores the relevance of GBF Target 3 (CBD 2022), which calls for expanding protected areas. However, our results underscore that expanding protected areas alone is insufficient. To effectively halt and reverse biodiversity loss, it is equally crucial to maintain and enhance habitat quality, as outlined in GBF Target 2 (CBD 2022), which calls for the restoration and effective management of at least 30% of terrestrial and marine ecosystems. Without these concerted efforts, the area required for biodiversity conservation will continue to escalate due to ongoing habitat degradation.

### 3.2 Protected Areas and Biodiversity: Stabilization Within Protected Areas and Decline in Unprotected Landscapes

To evaluate long‐term biodiversity outcomes under different management regimes, we examined temporal changes in species accumulation curves inside and outside protected areas. We hypothesised that (1) protected areas would exhibit more stable temporal trajectories due to sustained conservation management, whereas (2) unprotected areas would show continued declines in native plant biodiversity as a consequence of ongoing environmental pressures and the absence of formal conservation measures.

**FIGURE 2:**
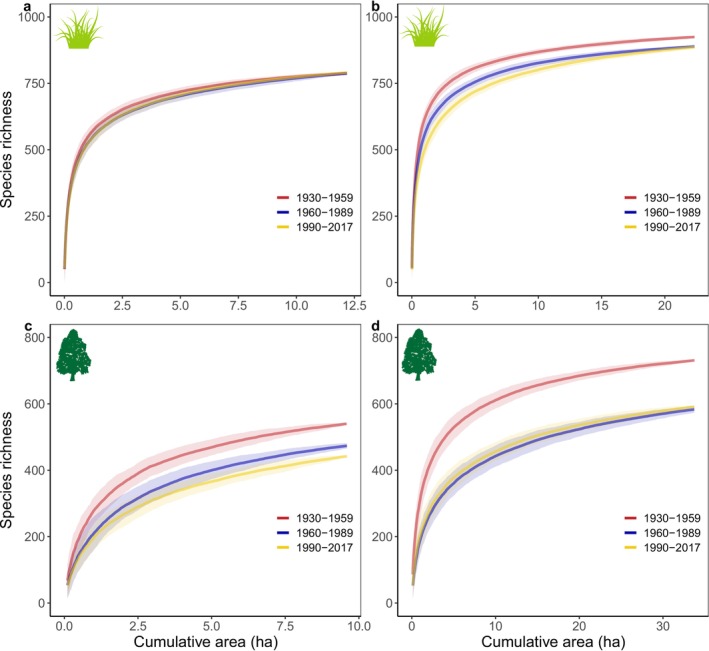
Species‐accumulation curves of native species richness in relation to cumulative sampled area from disjunct plots inside and outside protected areas in the Netherlands. (a, b) Species accumulation curves for grasslands inside (a) and outside (b) protected areas. (c, d) Species accumulation curves for forests inside (c) and outside (d) protected areas. Shaded areas indicate 95% confidence intervals.

Our findings partially support this hypothesis, demonstrating that protected areas play a key role in stabilizing species richness over time. Within protected areas, grassland species richness remained very similar (Figure 2a). This may highlight the effectiveness of conservation management in mitigating biodiversity decline, reinforcing the need to expand and enhance protected area management (Santangeli et al. 2023; Justin Nowakowski et al. 2023). It is worth noting that grassland species within protected areas even showed signs of very slight reversal of species‐accumulation trends (Figure 2a). This is an encouraging conservation outcome, as most studies suggest that while protected areas can slow biodiversity loss, they rarely reverse it (Santangeli et al. 2023; Jones 2024). In contrast, forest species exhibited greater fluctuations (Figure 2c), supporting the hypothesis that forest communities are more vulnerable to environmental change (Bertrand et al. 2016; Zhu et al. 2024). This suggests the need for stricter conservation measures and more targeted management strategies to prevent further accelerating biodiversity loss in forests.

In unprotected areas, species richness showed pronounced variability and marked historical declines in both grasslands and forests (Figure 2b,d), reflecting their exposure to land‐use change, fertilization, and other human pressures (Jaureguiberry et al. 2022; Díaz et al. 2019). Despite this, biodiversity stabilized after the 1990s (Figure 2b,d), indicating that broader landscape‐level interventions have had positive effects. Nevertheless, achieving biodiversity targets in these unprotected areas now requires more area than in the past, suggesting that recovery efforts need to be intensified to counterbalance previous losses.

## 4 Discussion

### 4.1 Drivers of Biodiversity Dynamics

Our findings provide critical insights into the long‐term dynamics of species‐accumulation patterns and the role of protected areas in conserving plant biodiversity. As human pressures on biodiversity intensify (Dirzo et al. 2014; Ceballos et al. 2015; Jaureguiberry et al. 2022; Keck et al. 2025), understanding these shifts is essential for tracking biodiversity trends, assessing conservation effectiveness, and informing policy interventions (Finderup Nielsen et al. 2019; Eichenberg et al. 2020; Jandt et al. 2022).

*Late‐20th‐century biodiversity decline (1960–1989)*: The substantial flattening of species‐accumulation curves after the 1930–1959 coincides with a period of intensive agricultural intensification and landscape restructuring in the Netherlands. Post‐war food security initiatives and the Green Revolution triggered widespread landscape modification (van Veen et al. 2008; van Strien et al. 2019). These transformations caused severe losses of semi‐natural ecosystems, with up to 74% of semi‐natural grasslands disappearing before 1990 (van Strien et al. 2019).

Simultaneously, nitrogen deposition increased sharply, from roughly 10–15 kg N ha^−1^ year^−1^ in the 1950s to 25–35 kg N ha^−1^ year^−1^ by the 1980s, exceeding critical loads for nutrient‐poor habitats by up to a factor of three (Bobbink and Hettelingh 2011; van der Hagen et al. 2023). This excessive nutrient input promoted competitive exclusion and biotic homogenization (Carvalheiro et al. 2013).

Importantly, these environmental pressures unfolded before the introduction of strong conservation legislation. The combination of agricultural intensification, eutrophication, and limited legal protection likely underlies the pronounced late‐20th‐century declines reflected in the species‐accumulation curves (van Veen et al. 2008).

*Mitigation and partial recovery (1990–2017)*: The slowdown in biodiversity decline and partial rebound in species‐accumulation patterns since the 1990s likely reflect major policy reforms and advances in conservation management. Emission regulations reduced nitrogen deposition by about 50% (CLO 2025a; van der Hagen et al. 2023). Although deposition remains above critical thresholds in some ecosystems, this reduction has eased competitive dominance by nitrophilous species and created conditions for partial vegetation recovery (van Veen et al. 2008).

During the same period, the EU Habitats Directive and Natura 2000 introduced legally binding habitat protection and active management obligations (Trochet and Schmeller 2013). The Netherlands expanded protected areas to more than 160 Natura 2000 sites, covering over 5500 km^2^ terrestrial areas (https://www.eea.europa.eu/en/analysis/maps‐and‐charts/natura‐2000‐barometer‐dashboards). Complementing these measures, the National Ecological Network (NEN) improved habitat connectivity, ecological buffering and landscape coherence (CLO 2025b). By 2020, more than 600,000 ha were restored, reconnected, or brought under nature‐friendly management (CLO 2025b). These landscape‐scale efforts facilitated recolonisation from remnant source populations and reduced the impacts of habitat isolation.

Together, these environmental and policy improvements created conditions that slowed biodiversity loss and enabled early signs of recovery, although current ecosystems still fall short of historical baselines. Continued restoration and sustained reductions in environmental pressure remain essential for long‐term ecological recovery.

### 4.2 Lessons Learned for Future Biodiversity Conservation

Over the past century, species‐accumulation curves for vascular plants in the Netherlands have flattened since the 1930s, particularly in forests. Both the asymptotic richness (total species captured) and curve slopes have declined, indicating reduced species diversity and altered beta diversity patterns. These changes signal a decline in habitat quality and biodiversity loss. This trend underscores the need for enhanced conservation efforts to maintain biodiversity at historical levels (CBD 2022). It is encouraging that, while previous studies have reported continued biodiversity declines (Eichenberg et al. 2020; Jandt et al. 2022), our results reveal signs of species recovery, suggesting that with sustained efforts, biodiversity decline can be mitigated and even partially reverted, though reaching historical levels remains challenging. However, sustaining and amplifying these gains will require ongoing investments in habitat restoration and management (Bell‐James and Watson 2025). Without such efforts, biodiversity is likely to decline further, requiring increasingly larger areas to protect the same number of species (Leadley et al. 2022). Note that maintenance and restoration of plant richness and diversity will have a positive cascading effect on invertebrate diversity, which has also declined substantially (Hallmann et al. 2017; Biesmeijer et al. 2006).

Our study also highlights the critical role of protected areas in stabilising species‐accumulation patterns, with biodiversity trends remaining more resilient within protected sites. This resilience demonstrates the effectiveness of protected areas in mitigating species loss and sustaining biodiversity despite broader environmental shifts (Wauchope et al. 2022). However, conservation benefits vary across ecosystems, with forest biodiversity showing weaker responses to protection compared to grassland species. Ineffective management within protected areas could further undermine conservation outcomes (Jones et al. 2018; Tollefson 2021; Appleton et al. 2022), particularly for forests, which remain highly vulnerable to various threats (Song and Zhu 2024). Strengthening conservation strategies is, therefore, essential to safeguard biodiversity in the face of ongoing environmental change.

Despite the challenges, our findings highlight that biodiversity recovery is possible with sustained conservation efforts. Although species‐accumulation patterns have yet to return to historical baselines, the observed stabilization and localized recoveries demonstrate that conservation interventions are yielding tangible benefits. Recognizing and building upon these successes is crucial for fostering continued investment in biodiversity conservation (Leung et al. 2020). Ongoing conservation and habitat restoration efforts are reversing the biodiversity trend, underscoring the importance of comprehensive monitoring to record successes and learn from failures, thus fostering evidence‐based biodiversity conservation (Grant et al. 2019; Moor et al. 2022).

### 4.3 Implications for Post‐2020 Conservation Strategies

Our findings have important implications for future conservation strategies, particularly within the post‐2020 biodiversity framework (CBD 2022; Ministry of LVVN 2025; EU 2024). We highlight two key insights: (1) species‐accumulation curves have changed over time, with declining total richness, local diversity and altered beta diversity patterns indicating biodiversity loss, particularly severe in forests and (2) protected areas are effective for stabilizing biodiversity, but guaranteeing long‐term plant conservation requires both strategic expansion of protected areas and complementary habitat restoration and management across the wider landscape, beyond protected area boundaries. These findings reinforce the urgency of meeting GBF Target 3 to protect at least 30% of land and sea by 2030, but also emphasize the critical importance of GBF Target 2, which calls for large‐scale habitat restoration beyond protected areas (CBD 2022).

The observed shifts in species‐accumulation patterns, evident from reduced total richness, local diversity and altered spatial turnover, highlight the need for ambitious yet strategic conservation policies. Expanding protected areas alone will not be sufficient to ‘bend the curve’ of biodiversity loss. Enhancing habitat quality through targeted management, both inside and outside protected areas, is equally crucial. Initiatives such as the Netherlands' Basic Quality of Nature program (https://toolbox‐all4biodiversity.nl/basiskwaliteit‐natuur‐startpagina) and the EU's Nature Restoration Regulation (https://environment.ec.europa.eu/topics/nature‐and‐biodiversity/nature‐restoration‐regulation_en) exemplify this integrated approach. Without such measures, conservation efforts risk becoming overly reliant on continually expanding protected areas, a strategy that is neither sustainable nor feasible in the long term. This is because landscapes surrounding protected areas are often the primary sources of environmental pressures driving biodiversity loss within reserves (IPBES 2019), while also serving as potential supplementary habitats. Without addressing these broader environmental pressures, efforts to halt or reverse biodiversity decline through protected areas alone will remain severely constrained.

Our results also underscore the vulnerability of forest ecosystems, which exhibit greater declines than grasslands. Forest biodiversity faces compounded threats from habitat degradation, climate change, and anthropogenic pressures (Bertrand et al. 2016; Zhu et al. 2024; Hoang and Kanemoto 2021; Sanczuk et al. 2024), necessitating stricter conservation interventions. Addressing these challenges requires a proactive, ecosystem‐specific approach that integrates habitat conservation, targeted restoration, enhanced connectivity, and resilience‐building strategies that take climate changes into account.

Finally, long‐term ecological monitoring is indispensable for assessing conservation effectiveness. Datasets such as the vegetation‐plot records analysed in this study provide a crucial evidence base for refining conservation policies and adapting management strategies. Strengthening long‐term monitoring frameworks will be key to ensuring that conservation interventions remain data‐driven, adaptive, and effective in reversing biodiversity decline.

## 5 Limitations and Conclusion

Despite drawing on an exceptional dataset, over 600,000 vegetation plots spanning nearly a century, some limitations remain. Species accumulation curves reflect richness gained by aggregating spatially disjunct plots, not contiguous areas as would be typical in protected area design (Smith 2010). Although we rigorously standardized plot spatial distributions, sizes, and sampling intensities across time periods, the accumulated ‘area’ in our curves represents the sum of many small, scattered plots rather than consolidated landscape blocks. Nevertheless, the strong spatial standardization implemented here ensures that temporal trends in species‐accumulation patterns reflect genuine ecological changes rather than sampling artefacts. The observed declines in maximum richness (curve asymptotes) and changes in curve shapes provide robust evidence of biodiversity state changes over the past century.

Second, multiple environmental drivers changed simultaneously, limiting our ability to attribute recovery to specific policies or environmental changes. Lack of plot‐level historical environmental data further constrains mechanistic inference. Future work integrating vegetation plots with spatial data on policy implementation, restoration interventions, nitrogen deposition, hydrology, and land management would allow stronger causal assessments.

Despite these constraints, our study provides empirical evidence of long‐term shifts in species‐accumulation patterns, with profound implications for biodiversity conservation. The increasing habitat requirements needed to sustain species underscore the challenges of reversing biodiversity loss. However, the relative stability of species‐accumulation patterns within protected areas demonstrates their effectiveness in mitigating species declines. Moreover, early signs of biodiversity recovery in some landscapes highlight the potential for conservation strategies to yield positive outcomes when implemented at scale.

To effectively safeguard biodiversity, conservation efforts must extend beyond protected areas to include comprehensive habitat restoration and management, particularly in forest ecosystems. Integrating these insights into global conservation frameworks will be essential for ensuring the persistence of plant biodiversity in the face of mounting environmental pressures.

## Author Contributions

K.P., J.C.B. and G.R.S. conceived the idea and designed the study. K.P. collected the data. K.P. performed the analyses and drafted the figures. K.P. contributed to the data management and maintenance. K.P. wrote the first draft. All authors reviewed and edited the manuscript. All authors contributed to the discussion of contents. All authors contributed to the design of the methodology. L.M., J.C.B. and G.R.S. contributed equally to this research.

## Conflicts of Interest

The authors declare no conflicts of interest.

## Supporting information


**Figure S1:** Workflow diagram showing data processing, spatial standardization, and analysis of temporal changes in species‐area relationships.
**Figure S2:** Spatial distribution of vegetation plots across the Netherlands following spatial standardization for (a) grasslands and (b) forests. Maps show plot locations (coloured dots) for three time periods (left to right: 1930–1959, 1960–1989 and 1990–2017), with each plot coloured by its assigned spatial cluster. Plus symbols (+) indicate the centroids of the k‐means spatial clusters. The standardized dataset exhibits comparable geographic coverage, plot density, and spatial configuration across periods (Figures S3 and S4), ensuring that temporal shifts in species accumulation curves represent genuine biodiversity changes rather than sampling artefacts.
**Figure S3:** Sample size per spatial region across time periods for (a) grasslands and (b) forests. Barplots show the number of plots sampled in each of the spatial regions (*x*‐axis) for the three time periods (coloured bars: red = 1930–1959; blue = 1960–1989 and yellow = 1990–2017). The constrained sampling ensures equal plot counts per region across all three periods. Only regions with data in all three periods (common regions) are retained in the standardized dataset, ensuring balanced temporal comparisons.
**Figure S4:** Validation of spatial standardization quality for (a–c) grasslands and (d–f) forests. Three key metrics demonstrate the standardization quality across time periods (1930–1959, 1960–1989 and 1990–2017). Left panels (a, d): Mean nearest‐neighbour distance across spatial regions, showing comparable inter‐plot spacing across periods after standardization. Middle panels (b, e): Mean plot size across spatial regions, demonstrating similar area distributions across periods. Right panels (c, f): Total sample size per time period, showing equal plot counts achieved through standardization (grasslands: 12,856 plots, forests: 1764 plots). Boxplots display variation across regions. Kruskal‐Wallis tests were used to assess whether the three time periods were statistically indistinguishable in plot spacing and plot size. In cases where significant differences emerged, we compared these patterns to the temporal trends in species‐accumulation curves. If differences in plot spacing or plot size did not align with the temporal pattern in species‐accumulation curves, the observed shifts in species accumulation curves were interpreted as genuine biodiversity changes rather than sampling artefacts. Significance letters indicate statistical differences among periods, with a denoting lower values and b denoting higher values.
**Figure S5:** Spatial distribution of vegetation plots across the Netherlands following spatial standardization within protected areas (a, b) and outside protected areas (c, d). Panels show (a) grasslands within protected areas, (b) forests within protected areas, (c) grasslands outside protected areas and (d) forests outside protected areas. Within each panel, maps display three time periods from left to right: 1930–1959, 1960–1989 and 1990–2017. Individual plots are shown as coloured dots, with colours indicating assignment to k‐means spatial clusters (cluster numbers vary by habitat‐protection combination). Plus symbols (+) mark cluster centroids. The standardized dataset exhibits comparable geographic coverage, plot density, and spatial configuration across periods within each habitat‐protection category (Figures S6 and S7), ensuring that temporal shifts in species accumulation curves represent genuine biodiversity changes rather than sampling artefacts.
**Figure S6:** Sample size per spatial region across time periods within protected areas (a, b) and outside protected areas (c, d). Panels show (a) grasslands within protected areas, (b) forests within protected areas, (c) grasslands outside protected areas and (d) forests outside protected areas. Within each panel, barplots show the number of plots sampled in each spatial region (*x*‐axis) for the three time periods (coloured bars: red = 1930–1959; blue = 1960–1989 and yellow = 1990–2017). The constrained sampling ensures equal plot counts per region across all three periods. Only regions with data in all three periods (common regions) are retained in the standardized dataset, ensuring balanced temporal comparisons.
**Figure S7:** Validation of spatial standardization quality within protected areas (a–f) and outside protected areas (g–l). Panels show three metrics for four habitat‐protection combinations: (a–c) grasslands within protected areas, (d–f) forests within protected areas, (g–i) grasslands outside protected areas and (j–l) forests outside protected areas. Within each row, three key metrics demonstrate standardization quality across time periods (1930–1959, 1960–1989 and 1990–2017). Left column (a, d, g, j): Mean nearest‐neighbour distance per spatial region, showing comparable inter‐plot spacing across periods. Middle column (b, e, h, k): Mean plot size per spatial region, demonstrating similar area distributions across periods. Right column (c, f, i, l): Total sample size per time period, showing equal plot counts achieved through standardization. Boxplots display variation across regions. Kruskal‐Wallis tests were used to assess whether the three time periods were statistically indistinguishable in plot spacing and plot size. In cases where significant differences emerged, we compared these patterns to the temporal trends in species‐area curves. If differences in plot spacing or plot size did not align with the temporal pattern in species‐area curves, the observed shifts in species accumulation curves were interpreted as genuine biodiversity changes rather than sampling artefacts. Significance letters indicate statistical differences among periods, with a denoting lower values and b denoting higher values.
**Figure S8:** Diversity patterns of plots over time. (a, b) Plot‐level alpha diversity. Species richness distributions for (a) grasslands and (b) forests across time periods. Boxplots show quartiles; letters indicate Kruskal‐Wallis significance groups with Bonferroni correction (*p* < 0.05). (c, d) Distance‐decay of community dissimilarity. Jaccard dissimilarity versus geographic distance for (c) grasslands and (d) forests. Dashed lines: linear regression with 95% CI. (e, f) Distance‐decay parameters. Regression slopes for (e) grasslands and (f) forests. Bars: single coefficient per period; error bars: ±SE from regression; letters: significance groups (*p* < 0.05).

## Data Availability

The vegetation plot data used in this study is sourced from the Dutch Vegetation Database (Hennekens 2018). Natura 2000 protected areas data are provided by the European Commission (https://www.eea.europa.eu/). The Red List of Vascular Plants of the Netherlands is sourced from Sparrius et al. (2014). Analyses were conducted using R (version 4.3.1) and R studio (version 2023.12.1.402), with detailed methodologies provided in the Methods section. The complete R code and processed data required to reproduce all analyses and figures are available via Zenodo https://doi.org/10.5281/zenodo.19029884 (Pan et al. 2026).
