# Supplementary material for: A Century of Shifting Native Species‐Accumulation Curves Reveals Long‐Term Biodiversity Loss
Source: Ecol Lett. 2026 Jun 15;29(6):e70429. doi: 10.1111/ele.70429 (PMC13267914; doi:10.1111/ele.70429)
Supplement: Supplementary file 1 — Figure S1: Workflow diagram showing data processing, spatial standardization, and analysis of temporal changes in species‐area relationships. Figure S2: Spatial distribution of vegetation plots across the Netherlands following spatial standardization for (a) grasslands and (b) forests. Maps show plot locations (coloured dots) for three time periods (left to right: 1930–1959, 1960–1989 and 1990–2017), with each plot coloured by its assigned spatial cluster. Plus symbols (+) indicate the centroids of the k‐means spatial clusters. The standardized dataset exhibits comparable geographic coverage, plot density, and spatial configuration across periods (Figures S3 and S4), ensuring that temporal shifts in species accumulation curves represent genuine biodiversity changes rather than sampling artefacts. Figure S3: Sample size per spatial region across time periods for (a) grasslands and (b) forests. Barplots show the number of plots sampled in each of the spatial regions (x‐axis) for the three time periods (coloured bars: red = 1930–1959; blue = 1960–1989 and yellow = 1990–2017). The constrained sampling ensures equal plot counts per region across all three periods. Only regions with data in all three periods (common regions) are retained in the standardized dataset, ensuring balanced temporal comparisons. Figure S4: Validation of spatial standardization quality for (a–c) grasslands and (d–f) forests. Three key metrics demonstrate the standardization quality across time periods (1930–1959, 1960–1989 and 1990–2017). Left panels (a, d): Mean nearest‐neighbour distance across spatial regions, showing comparable inter‐plot spacing across periods after standardization. Middle panels (b, e): Mean plot size across spatial regions, demonstrating similar area distributions across periods. Right panels (c, f): Total sample size per time period, showing equal plot counts achieved through standardization (grasslands: 12,856 plots, forests: 1764 plots). Boxplots display variation acros [file ELE-29-0-s001.docx]

**Supplementary Materials**

**A century of shifting native species-area curves reveals long-term biodiversity loss**

Kaixuan Pan^1,2✉️^, Leon Marshall^1^, Jacobus C. Biesmeijer^1,2^ & Geert R. de Snoo^2,3^

1 *Naturalis Biodiversity Center, Leiden, The Netherlands*

2 *Institute of Environmental Sciences, Leiden University, Leiden, The Netherlands*

3 *Netherlands Institute of Ecology (NIOO-KNAW), Wageningen, The Netherlands*

✉️Corresponding author: [kaixuan.pan@naturalis.nl](mailto:kaixuan.pan@naturalis.nl) (K.P)

Other co-authors’ emails: [leon.marshall@naturalis.nl](mailto:leon.marshall@naturalis.nl) (L.M), [koos.biesmeijer@naturalis.nl](mailto:koos.biesmeijer@naturalis.nl) (J.C.B) and [geert.de.snoo@knaw.nl](mailto:geert.de.snoo@knaw.nl) (G.R.d.S)

**Table of contents:**

Supplementary methods

Figure S1: Workflow diagram showing data processing, spatial standardization, and analysis of temporal changes in species-area relationships.

Figure S2: Spatial distribution of vegetation plots across the Netherlands following spatial standardization for (a) grasslands and (b) forests.

Figure S3: Sample size per spatial region across time periods for (a) grasslands and (b) forests.

Figure S4: Validation of spatial standardization quality for (a-c) grasslands and (d-f) forests.

Figure S5: Spatial distribution of vegetation plots across the Netherlands following spatial standardization within protected areas (a-b) and outside protected areas (c-d).

Figure S6: Sample size per spatial region across time periods within protected areas (a-b) and outside protected areas (c-d).

Figure S7: Validation of spatial standardization quality within protected areas (a-f) and outside protected areas (g-l).

### **Supplementary methods**

To investigate mechanisms underlying species-accumulation curve shifts, we conducted complementary analyses using the same standardized plots as SAC rarefaction to ensure consistency.

***Distance-decay beta diversity analysis*.** We quantified spatial turnover by examining community dissimilarity versus geographic distance. For each period, we randomly sampled 2,000 plot pairs, calculated pairwise Jaccard dissimilarity, and geographic distance (Euclidean, km). We fitted linear regressions (dissimilarity ~ distance) to quantify spatial turnover rates (slopes). Steeper slopes indicate faster community turnover over distance (spatial segregation); shallower slopes indicate slower turnover (homogenization). This directly tests mechanism of beta diversity pattern changes.

***Alpha diversity distribution*.** We tested whether plot-level species richness changed significantly across periods using Kruskal-Wallis tests with Bonferroni correction for multiple comparisons. Significance groups were assigned using the agricolae package in R. This provides statistical validation of alpha changes and complements beta partitioning.

Analyses were conducted separately for grassland and forest ecosystems to detect ecosystem-specific patterns.


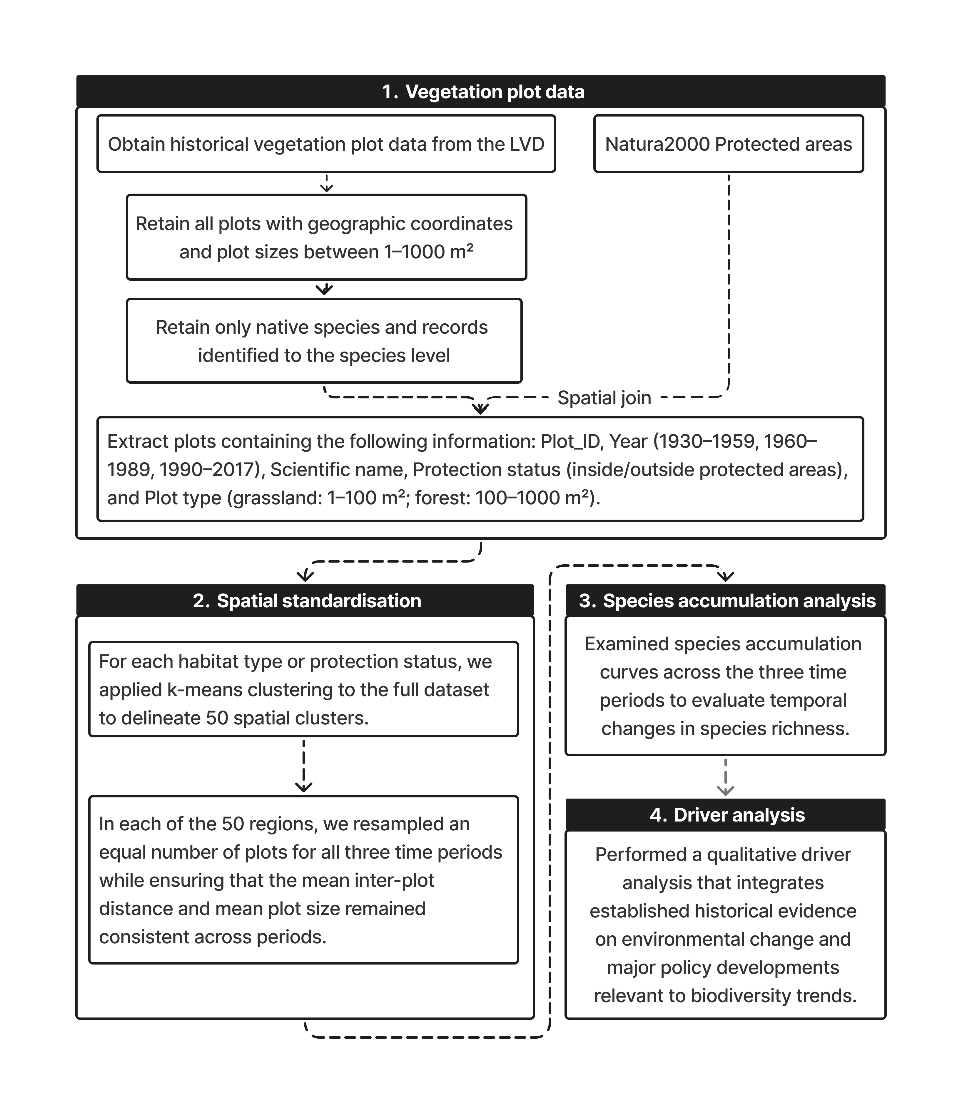


**Figure S1: Workflow diagram showing data processing, spatial standardization, and analysis of temporal changes in species-area relationships.**


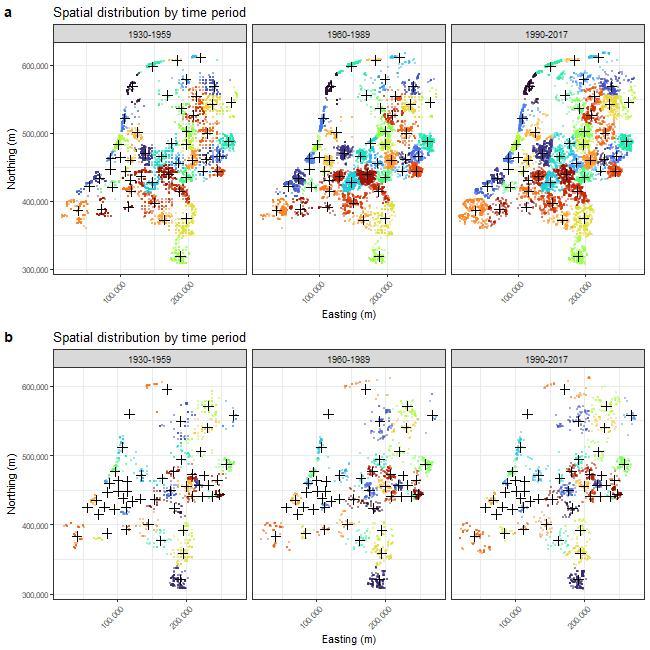


**Figure S2: Spatial distribution of vegetation plots across the Netherlands following spatial standardization for (a) grasslands and (b) forests.** Maps show plot locations (colored dots) for three time periods (left to right: 1930-1959, 1960-1989, 1990-2017), with each plot colored by its assigned spatial cluster. Plus symbols (+) indicate the centroids of the k-means spatial clusters. The standardized dataset exhibits comparable geographic coverage, plot density, and spatial configuration across periods (Figure S3, Figure S4), ensuring that temporal shifts in species accumulation curves represent genuine biodiversity changes rather than sampling artifacts.


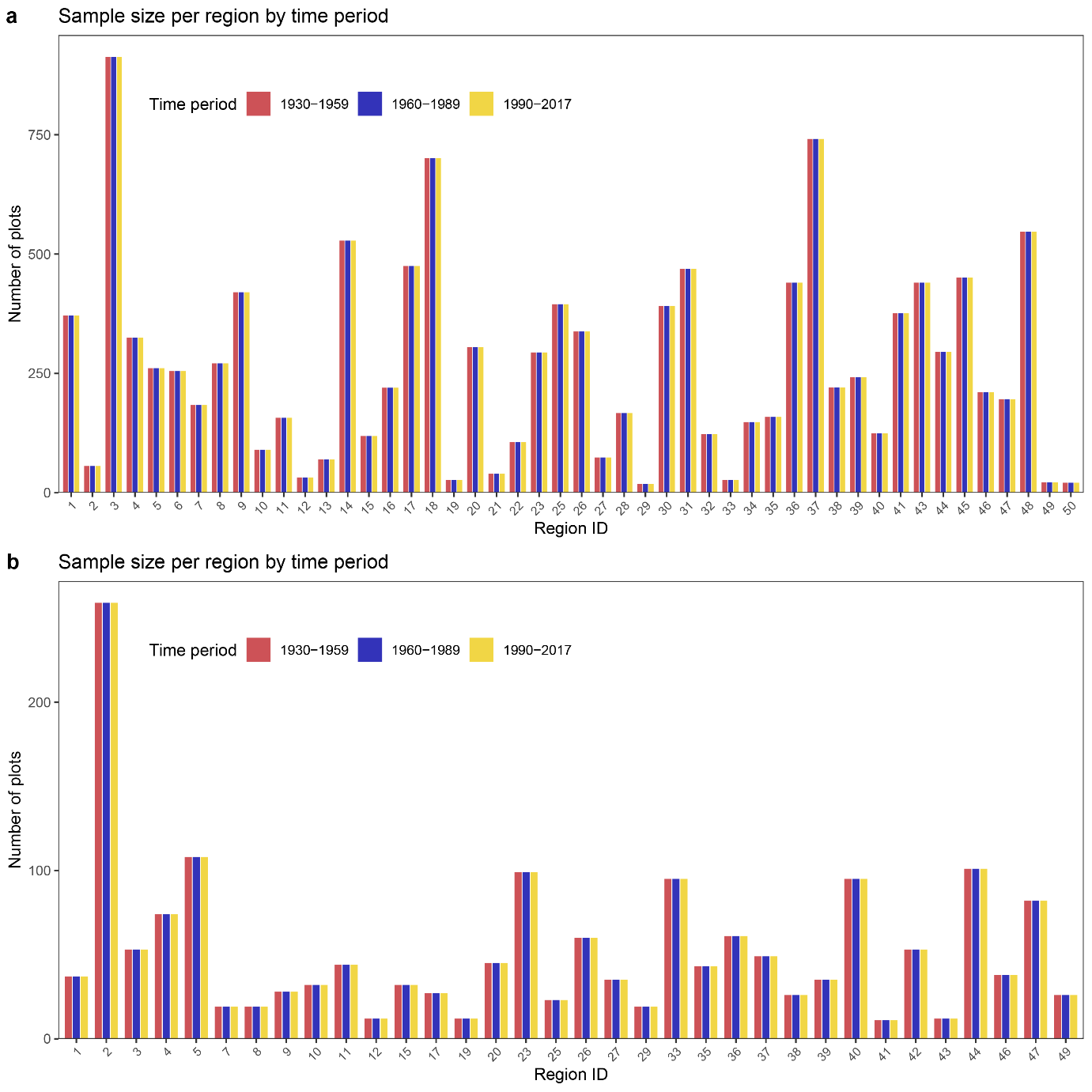


**Figure S3: Sample size per spatial region across time periods for (a) grasslands and (b) forests.** Barplots show the number of plots sampled in each of the spatial regions (x-axis) for the three time periods (colored bars: red = 1930-1959; blue = 1960-1989; yellow = 1990-2017). The constrained sampling ensures equal plot counts per region across all three periods. Only regions with data in all three periods (common regions) are retained in the standardized dataset, ensuring balanced temporal comparisons.


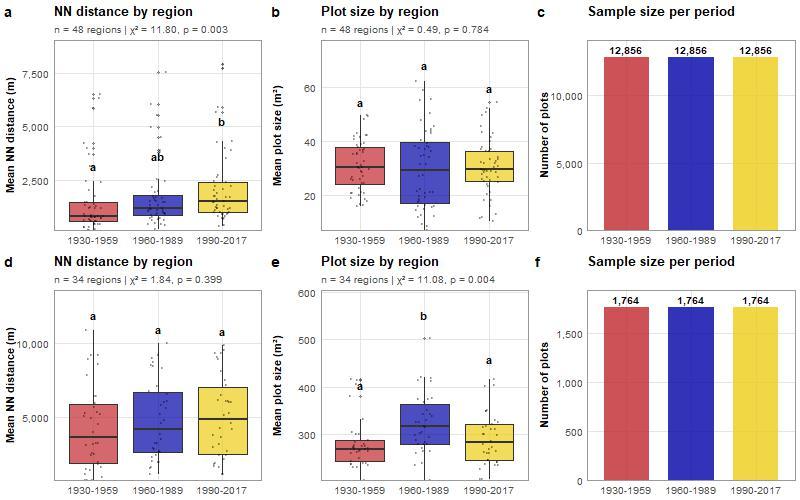


**Figure S4: Validation of spatial standardization quality for (a-c) grasslands and (d-f) forests.** Three key metrics demonstrate the standardization quality across time periods (1930-1959, 1960-1989, 1990-2017). **Left panels (a, d)**: Mean nearest-neighbor distance across spatial regions, showing comparable inter-plot spacing across periods after standardization. **Middle panels (b, e)**: Mean plot size across spatial regions, demonstrating similar area distributions across periods. **Right panels (c, f)**: Total sample size per time period, showing equal plot counts achieved through standardization (grasslands: 12,856 plots, forests: 1,764 plots). Boxplots display variation across regions. Kruskal-Wallis tests were used to assess whether the three time periods were statistically indistinguishable in plot spacing and plot size. In cases where significant differences emerged, we compared these patterns to the temporal trends in species-accumulation curves. If differences in plot spacing or plot size did not align with the temporal pattern in species-accumulation curves, the observed shifts in species accumulation curves were interpreted as genuine biodiversity changes rather than sampling artefacts. Significance letters indicate statistical differences among periods, with a denoting lower values and b denoting higher values.


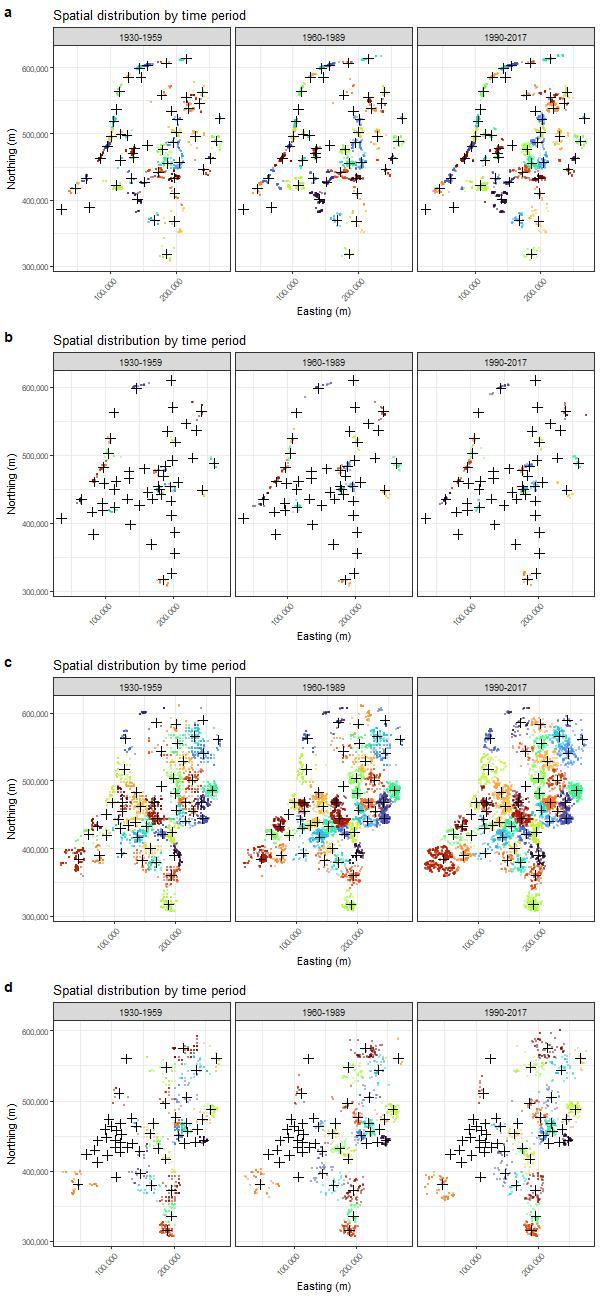


**Figure S5: Spatial distribution of vegetation plots across the Netherlands following spatial standardization within protected areas (a-b) and outside protected areas (c-d).** Panels show (**a**) grasslands within protected areas, (**b**) forests within protected areas, (**c**) grasslands outside protected areas, and (**d**) forests outside protected areas. Within each panel, maps display three time periods from left to right: 1930-1959, 1960-1989, and 1990-2017. Individual plots are shown as colored dots, with colors indicating assignment to k-means spatial clusters (cluster numbers vary by habitat-protection combination). Plus symbols (+) mark cluster centroids. The standardized dataset exhibits comparable geographic coverage, plot density, and spatial configuration across periods within each habitat-protection category (Figure S6, Figure S7), ensuring that temporal shifts in species accumulation curves represent genuine biodiversity changes rather than sampling artifacts.


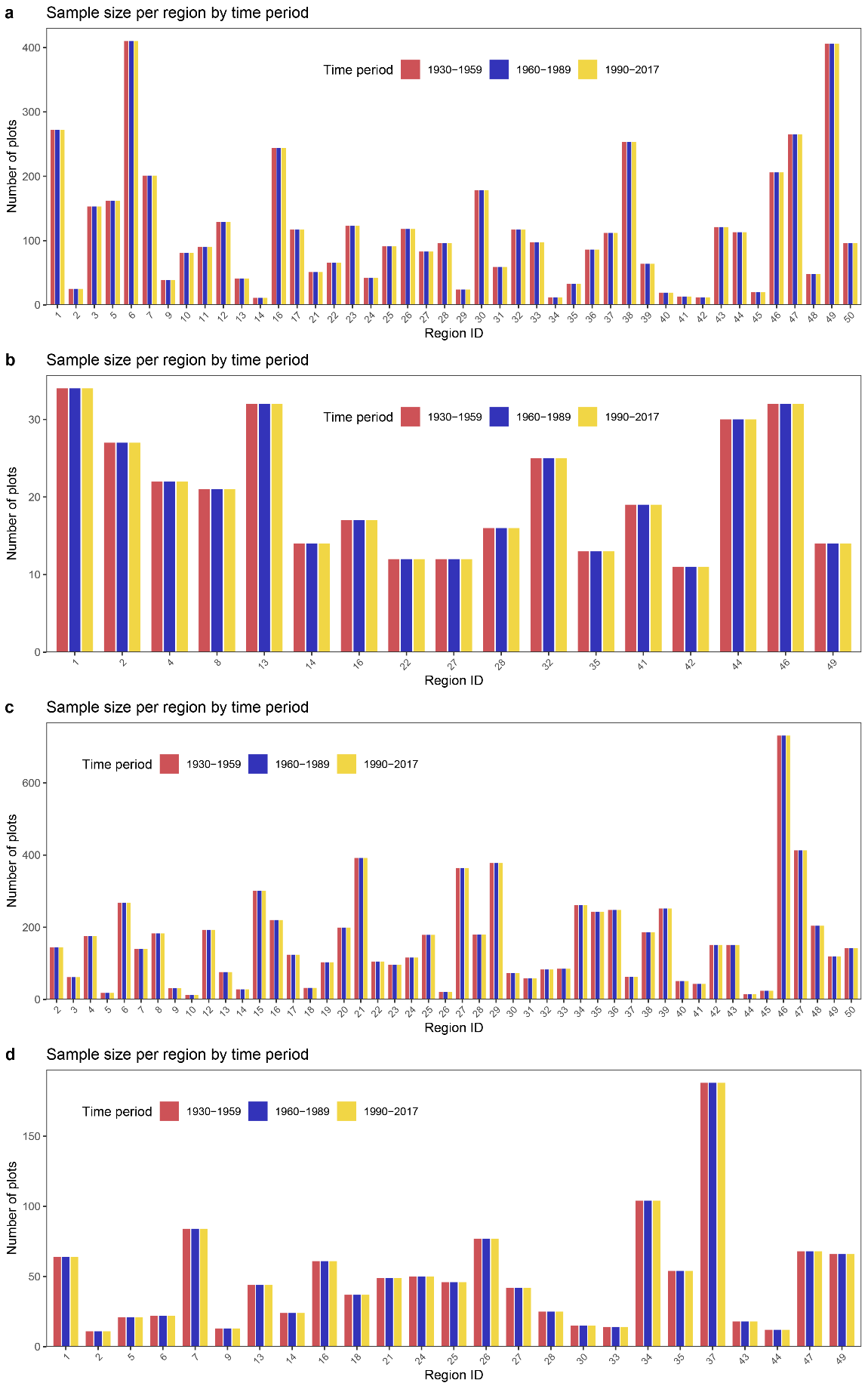


**Figure S6: Sample size per spatial region across time periods within protected areas (a-b) and outside protected areas (c-d).** Panels show (**a**) grasslands within protected areas, (**b**) forests within protected areas, (c) grasslands outside protected areas, and (d) forests outside protected areas. Within each panel, barplots show the number of plots sampled in each spatial region (x-axis) for the three time periods (colored bars: red = 1930-1959; blue = 1960-1989; yellow = 1990-2017). The constrained sampling ensures equal plot counts per region across all three periods. Only regions with data in all three periods (common regions) are retained in the standardized dataset, ensuring balanced temporal comparisons.


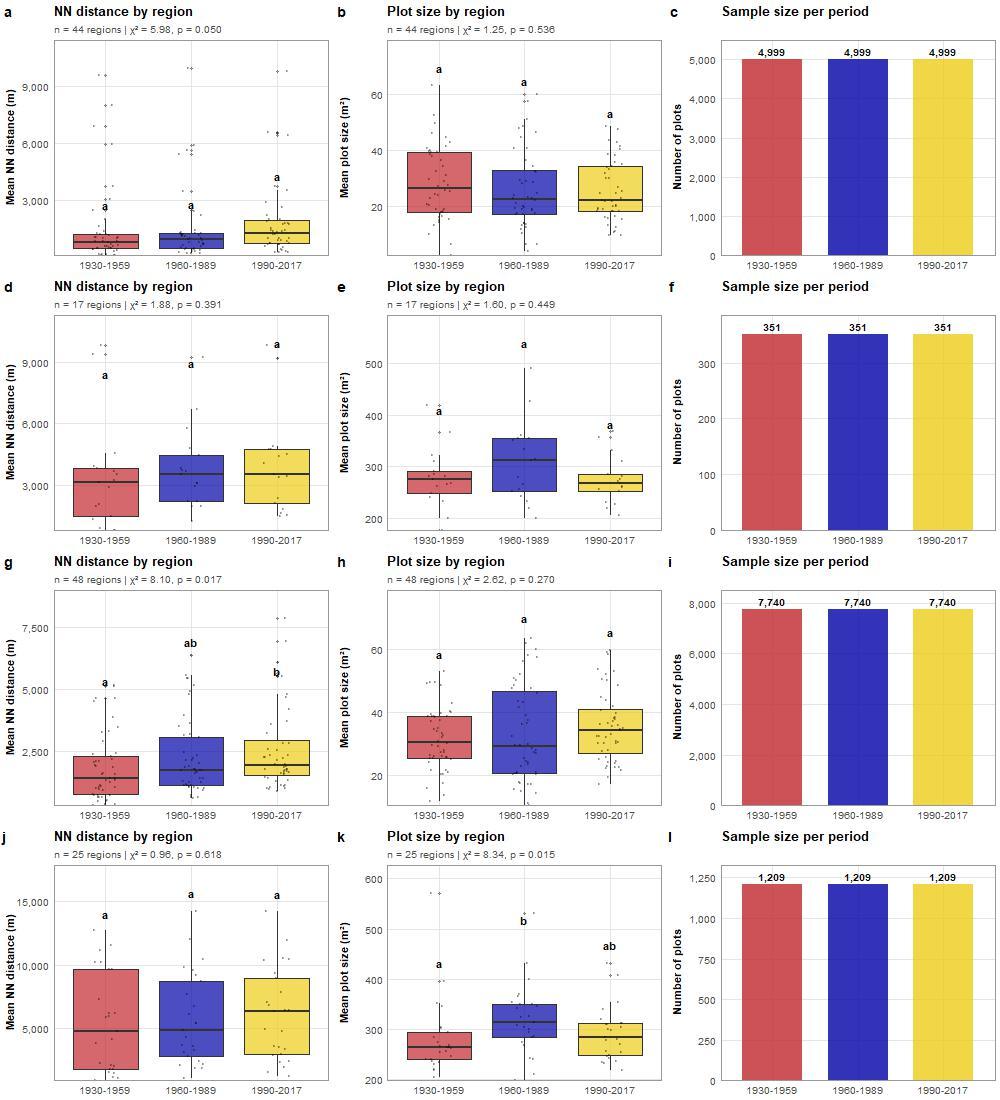


**Figure S7: Validation of spatial standardization quality within protected areas (a-f) and outside protected areas (g-l).** Panels show three metrics for four habitat-protection combinations: (**a-c**) grasslands within protected areas, (**d-f**) forests within protected areas, (**g-i**) grasslands outside protected areas, and (**j-l**) forests outside protected areas. Within each row, three key metrics demonstrate standardization quality across time periods (1930-1959, 1960-1989, 1990-2017). **Left column (a, d, g, j)**: Mean nearest-neighbor distance per spatial region, showing comparable inter-plot spacing across periods. **Middle column (b, e, h, k)**: Mean plot size per spatial region, demonstrating similar area distributions across periods. **Right column (c, f, i, l)**: Total sample size per time period, showing equal plot counts achieved through standardization. Boxplots display variation across regions. Kruskal-Wallis tests were used to assess whether the three time periods were statistically indistinguishable in plot spacing and plot size. In cases where significant differences emerged, we compared these patterns to the temporal trends in species-area curves. If differences in plot spacing or plot size did not align with the temporal pattern in species-area curves, the observed shifts in species accumulation curves were interpreted as genuine biodiversity changes rather than sampling artefacts. Significance letters indicate statistical differences among periods, with a denoting lower values and b denoting higher values.


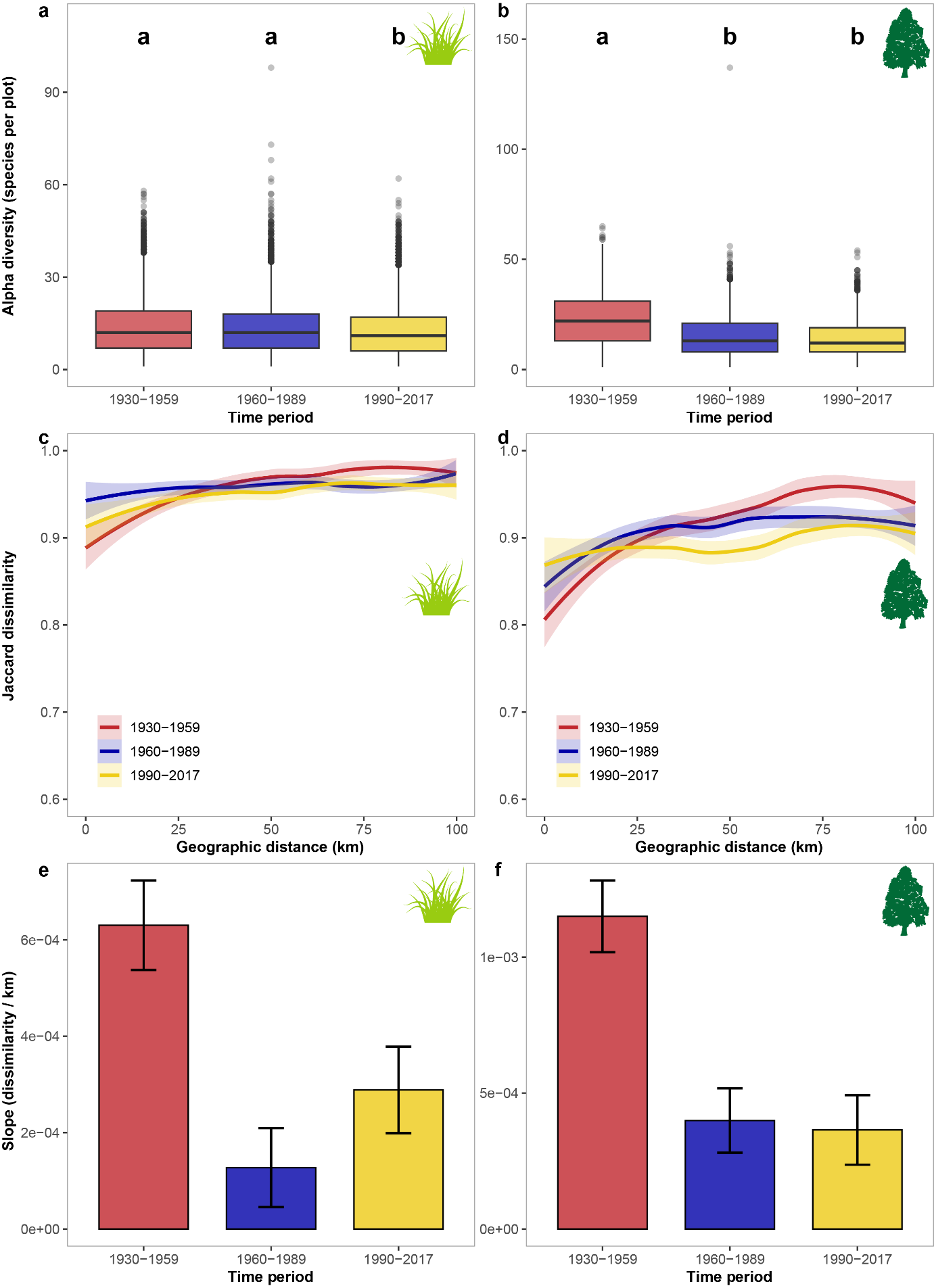


**Figure S8: Diversity patterns of plots over time.** (**a-b**) Plot-level alpha diversity. Species richness distributions for (**a**) grasslands and (**b**) forests across time periods. Boxplots show quartiles; letters indicate Kruskal-Wallis significance groups with Bonferroni correction (*p* < 0.05). (**c-d**) Distance-decay of community dissimilarity. Jaccard dissimilarity versus geographic distance for (**c**) grasslands and (**d**) forests. Dashed lines: linear regression with 95% CI. (**e-f**) Distance-decay parameters. Regression slopes for (**e**) grasslands and (**f**) forests. Bars: single coefficient per period; error bars: ±SE from regression; letters: significance groups (*p* < 0.05).
